# Polymer thin film-embedded metal oxide-modified electrochemical paper-based sensor for glycine detection†

**DOI:** 10.1039/d5ra01870f

**Published:** 2025-05-12

**Authors:** Zaheer Ahmad, Abdullah K. Alanazi

**Affiliations:** a Department of Chemistry, GPGC Timergara Dir Lower Khyber Pakhtunkhwa Pakistan saubanzaheer@gmail.com; b Higher Education, Archives and Libraries Department Government of Khyber Pakhtunkhwa Pakistan; c Department of Chemistry, College of Science, Taif University P.O. Box 11099 Taif 21944 Saudi Arabia

## Abstract

This work describes the design and selectivity of a simple electrochemical paper-based analytical sensor (ePAS) for the detection of an amino acid (glycine) using Whatman No. 1 filter paper as the substrate material. A simple wax fabrication method was used to create hydrophobic and hydrophilic channels to control the sample flow. For electrochemical analysis, the carbon and silver ink electrodes were printed on a paper. The working and counter electrodes were made from carbon ink, and the reference and connective pads were made of silver/silver chloride ink. A sensing material composed of methoxy poly(ethylene glycol)-*block*-poly(l-glutamic acid) and cadmium oxide quantum dots was utilized for the detection of glycine. The proposed ePAS exhibited potential electrocatalytic activity toward the oxidation of glycine. The electrochemical studies showed a higher sensitivity for glycine in a basic medium in a potential window of −0.4 to +0.6 V. The detection limit for glycine under optimized conditions achieved with this sensor was 75.8 μM, while the limit of quantification was 229.7 μM. The sensor showed high sensitivity and reproducibility for up to 25 cycles and directly quantified the amino acids in the samples. The sensor could be reused, making it more economical and environmentally friendly.

## Abstract 2

A simple electrochemical paper-based sensor (ePAS) using mPEG-*b*-PLG/CdO quantum dots enabled selective glycine detection. Wax-printed channels and carbon/silver electrodes made this green, reusable, and low-cost platform suitable for real samples.

## Graphical abstract

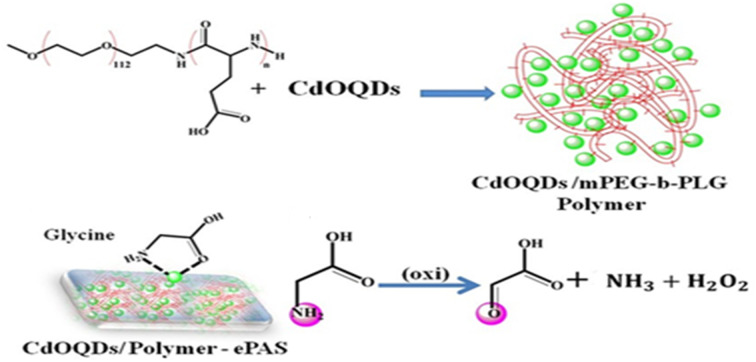

## Introduction

Observation of glycine as a biological indicator is of utmost importance owing to its involvement in numerous physiological processes, making it one of the key biomolecules in clinical assessments.^1–4^ Accordingly, more rapid, accurate and economical quantification techniques are required. The goal is to develop techniques that match or exceed the accuracy and reliability of current glycine detection benchmarks. Electrochemical paper-based sensors (ePASs) are among the most widely used sensors, which have gained attention in the field of point-of-care devices owing to its characteristics, such as being easy to handle, light in weight, economical and easy to transport. These sensors have great applications in food safety analysis, environmental monitoring, and diagnostic purposes.^5–9^ To get more rapid results during patient disease diagnosis, a new type of device called point-of-care devices were introduced. Such devices can be used to perform tests at or near the patient care site, which is known as bedside analysis. To enhance the efficiency of these devices, we should move one step further by replacing the old traditional ways with more advanced and modern point-of-care devices. Although such devices have been used since very old times, their applications have gained more interest in recent times.^10–12^ The introduction of nanotechnology in the field of sensors made it easy to fabricate such devices with more effectiveness. Nanotechnology has introduced a new way of developing point-of-care devices based on electrode systems, leading to electrochemical point-of-care devices. These devices, which are composed of screen-printed electrodes (SPEs), have unique characteristics of reproducibility, re-usability, and economical nature. The use of modern nanotechnology and fabrication techniques has made it possible to develop a sensor for point-of-care analysis that is simple, small and more economical.^13^ In recent times, the tool of nanotechnology combined with polymeric materials has resulted in more sophisticated sensors for the point-of-care analysis of various analytes, such as glucose, protein, nitrites, uric acid, lactate, ketones, and human IgG, Pb(ii), Fe(iii) and Au(iii).^14–16^

One of the most important organic materials is paper, which is made up of cellulose fibers and has the characteristics of porosity and permeability. The diameter of the paper ranges from 1 to 100 μm, and its pore size ranges from 1 to 10 μm. The hydrophilic nature of these materials makes them good flow-based substances.^17–19^ The paper-based sensor offers many advantages, including biological biocompatibility, biodegradability, low cost and easy accessibility.^20^ The problems associated with other sensing materials, like unequal distribution of samples, larger sample sizes, and danger associated with sample discard, can be easily solved using paper-based materials. Paper-based material samples can be easily distributed in many channels without any extra requirement for suction pumps.^21^ The development of paper-based point-of-care devices is very easy and cheaper. The first paper-based point-of-care device was reported by a group from Harvard University.^22^ The group used a photolithographic method for developing chips in which hydrophobic walls were created on hydrophilic cellulose paper, leading to the formation of small-sized channels.^23^ In another study, Yagoda *et al.* introduced wax fabrication on filter paper in 1937.^17^ In recent research, many lightweight paper-based biomarker analyses have been used. The wax-structuring method offers several advantages, including easy fabrication, speed, low cost, simplicity, environmental friendliness, non-toxicity and ease of disposal. In addition, this technique does not require a custom setup, ultraviolet (UV) lamps, and organic solvents.^24^ For practical applications in the field of assessing the amount of glucose or other metabolites, chips can be created using a printing method in which the electrode on the point of substance can be constructed.^25,26^ Based on previous approaches, this study developed high-resolution electrodes using conductive inks. Electrochemical analysis using different techniques, including amperometry, voltammetry and potentiometry, can be conducted using paper-based reference working and counter electrodes.^27,28^ Advancements in electrochemical analysis have led to improvements in printed electrodes, particularly in terms of reproducibility, inertness, low background current and a wide potential range. Such electrodes also offer additional benefits such as ease of operation, cost-effectiveness, small sample size requirements and suitability for on-site testing.^29,30^ These devices, in addition to other applications, show commercial potential as well.^31^ In the field of paper-based sensors, more research has been conducted. Complex and multi-analyte quantification can be performed by managing the path dimensions and sample flow time of the paper-based devices.^32^ Due to the involvement of heavy metals in the sample interference, this interference can be addressed by the introduction of new sensing materials based on maximization of electrode selectivity.^33^ The electrolytic capabilities of such sensors can be maximized by switching to nanosized materials. Modification of substrate surfaces using nano-based materials has gained more attention in recent years. Out of various efficient nanomaterials, quantum dots (QDs) have vast applications in the fields of catalysis, bioelectronics, optics, biomedical, chemical and biosensors.^34–36^ These vast applications of quantum dots are due to their large surface-to-volume ratio and physiochemical characteristics.

The polymer used in this study has several characteristic properties, including biodegradability, biocompatibility, high loading capability and responsiveness, making it suitable for applications in drug delivery, biosensing, and other related fields.^37–41^ Similarly, cadmium oxide quantum dots were also investigated.^42,43^

In this study, we present a novel sensing platform that integrates methoxy poly(ethylene glycol)-*block*-poly(l-glutamic acid) (mPEG-*b*-PLG) with cadmium oxide quantum dots (CdOQDs) to construct a highly sensitive paper-based electrochemical sensor. The tailored combination of a biodegradable, biocompatible polymer with responsive functionalities and the superior electrocatalytic activity of CdOQDs was designed to enhance the precision, stability, and applicability of the sensor. This innovative framework aims to bridge the gap between advanced nanomaterials and cost-effective sensing technologies, offering a promising approach for developing next-generation biosensing and diagnostic tools.

## Experimental section

### Reagents and apparatus

Buffers of potassium phosphate (98%) and potassium dihydrogen phosphate, alanine, acetone, formic acid and ammonia were obtained from Merck. Silver acetate and ferrous chloride were obtained from Sigma-Aldrich. Black ink and glue were purchased from a local market. All chemicals were used as received without further purification. For the preparation of different reagents, distilled water was used.

A cyclic voltammetry system (Model 600E) with a reference potentiostat (Gamry, Instruments Inc., Warminster PA, USA) was used for all kinds of electrochemical measurements. In all kinds of electrochemical analysis Autolab potentiostat/galvanostat type, PGSTAT 302N (Eco Chemie, Netherlands), was used with the GPES 4.9 software package.

### Reagents preparations

Different reagents in acetate buffer (pH ∼ 4) and phosphate buffer saline (PBS) solution (pH ∼ 7.4) were prepared as per protocol according to the desired concentrations as follows:

### Fabrication of the e-paper sensor

**Fig. 1:**
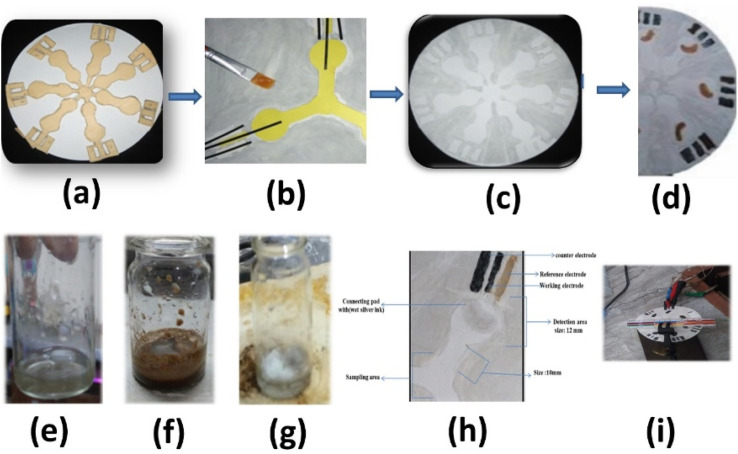
Steps for the fabrication: (a) paper with barrier for making hydrophilic and hydrophobic contact pads, (b) fabrication of paper with wax, (c) fabricated paper, (d) fabricated paper with conductive ink, (e) silver acetate dissolved in ammonia solution, (f) black precipitates formed upon addition of formic acid (g) prepared silver/silver chloride ink. (h) Electrodes. (i) Final shape.

To use filter paper as a substrate, many fabrication methods have been developed. Among all wax fabrication techniques, this technique is one of the best ever used. The moisture present in the filter paper was removed by soaking it in acetone, followed by drying it in an oven for 10 minutes at 70 °C. The wax printing technique was used to modify the surface of paper with wax by creating hydrophilic and hydrophobic channels. A paint brush was used to generate a channel shape on the paper. In another paper, channels were created using a stencil of a hard sheet. The stenciled sheet was then placed on the filter paper by temporary fixing. A uniform layer of melted wax was applied on both sides of the filter paper and left until complete drying. When complete drying occurred, a hard stencil sheet was detached from the filter paper, and hydrophobic channels were created. The same procedure was adopted in the case of printing electrodes. The complete setup is shown in Fig. 1.

The centre of the paper is the sampling area in which the sample can be placed. From the sampling area, the sample flows through the created channels to the detection zone, where the sensing materials and printed electrodes are incorporated. The size of this area is 12.0 mm with a circular shape. The channels were 10 mm long. To minimize the resistance between the working and reference electrodes, the distance is kept small. In common practice, the distance between the electrodes should not be more than 0.3 mm. In this study, the total area of the counter electrode was maintained at 15 mm^2^, while that of the working electrode was 2.6 mm^2^.

### Conductive ink for paper-based electrodes

Electrodes of various natures were designed using different inks. The working and counter electrodes were developed using carbon ink, whereas the reference electrode was developed using Ag/AgCl. Prussian blue was used as the carbon ink mediator.

To make a carbon electrode as shown in Fig. S1A,† various materials (1.0 g carbon, 1.0 mL ordinary glue, 1.0 mL ordinary black ink and 1.0 mg Prussian blue) were homogenously mixed in a test tube. The silver/silver chloride ink preparation procedure is illustrated in Fig. S1B.† In this procedure, the silver ink was first prepared by mixing 1.0 g of silver acetate with 2.5 mL of ammonia. The mixture was stirred until a clear solution was obtained, as shown in eqn (1).

1 2NH_3_ + CH_3_CO_2_Ag → [Ag(NH_3_)_2_]CH_3_CO_2_

Subsequently, formic acid was added dropwise until a black precipitate formed. The precipitate was then filtered out, and the clear solution was used as a silver/silver chloride connective ink, as shown in eqn (2).

2 HCOOH + NH_3_ → NH_4_HCOO

The ink was then dried on the paper surface, as described in eqn (3).

3 2[Ag(NH_3_)_2_]CH_3_CO_2_ + NH_4_HCOO → 2Ag + 2CH_3_COOH + 5NH_3_ + CO_2_

During this process, high precautions should be taken to avoid unusual situations. The detailed procedure for the preparation of Prussian blue is shown in Fig. S1C.†

### Synthesis of methoxy poly(ethylene glycol)-*block*-poly(l-glutamic acid) polymer

mPEG-*b*-PLG diblock copolymer was synthesized by polymerization of BLG-*b*-NCA as reported earlier.^39,44^ In the polymerization process, mPEG-NH_2_ was used as the initiator and the benzyl group as the de-protecting agent and for providing stability to the polymer. In the first step, 15.0 mM BLG–NCA and 0.6 mM mPEG-NH_2_ were dissolved in 70.0 mL dry DMF. After 3 days, the polymerization process was completed, and in the presence of diethyl ether, the precipitates of methoxy poly(ethylene glycol)-*b*-poly(γ-benzyl-l-glutamate) (mPEG-*b*-PBLG) block copolymers were developed. Subsequently, mPEG-*b*-PBLG was dissolved in dichloroacetic acid, and HBr/acetic acid was added. The mixture was then slowly stirred at 30 °C for 1 h, after which the final product was precipitated into excess diethyl ether. After drying under vacuum, a white solid was obtained (Scheme S1†).

### Cadmium oxide quantum dot (CdOQD) synthesis

Quantum dots of cadmium oxide were synthesized. For this purpose, 0.8 g of cadmium acetate was dissolved in distilled water and stirred at 40 °C. The ammonia solution was then added dropwise until the solution pH reached 8.0. The solution was then stirred for an additional 3 to 4 hours until a white precipitate was produced. The precipitate was then washed three or four times with distilled water and dried at 100 °C for 6 h. The solid was then ground into a fine powder and calcinated at 400 °C for 2 hours. The chemical reaction for the formation of cadmium oxide quantum dots is described by eqn (4).^42^

4 Cd(CH_3_COO)2·2H_2_O + 2NH_4_OH → Cd(OH)_2_ + 2H_2_O + 2CH_3_COONH_4_

5 Cd(OH)_2_ → CdO + H_2_O

### Modification of paper with polymer and CdOQDs material

To design a paper-based sensor for glycine detection purposes, the filter paper was modified with a methoxy poly(ethylene glycol)-*block*-poly(l-glutamic acid) (mPEG-*b*-PLG) polymer and cadmium oxide quantum dots (CdOQDs) to serve as the sensing matrix. The modified paper-based sensor provides an environment for analyte interactions that can be quantified through electrochemical responses. For the modification process, the polymer and CdOQDs were separately prepared in a phosphate buffer solution (PBS, 0.1 M, pH 7.4). First, the paper substrate (Whatman Grade 1 filter paper) was cut into strips (1 cm × 4 cm) and pre-treated by immersion in ethanol for 5 minutes to remove any surface impurities, followed by drying at room temperature. Next, 50 μL of mPEG-*b*-PLG polymer solution (1 mg mL^−1^ in PBS) was drop-cast onto the designated detection zone of each paper strip using a micropipette. The paper was allowed to dry at room temperature for 1 hour. Afterward, 50 μL of CdOQDs solution (0.5 mg mL^−1^ in PBS) was similarly applied to the same zone and allowed to dry for another hour to ensure firm attachment and proper interaction of the nanomaterials. After modification, the functionalized paper sensors were stored in a desiccator until use. During sensing, a small volume (typically 20–30 μL) of the sample was applied to the sample zone and allowed to migrate to the detection zone *via* capillary action. The electrochemical response was recorded once the sample reached the detection area using cyclic voltammetry (CV).^45^ The detailed working arrangement of the paper-based analytical device is shown in Fig. 1.

## Result and discussion

### Characterization of the synthesized polymer and cadmium oxide QDs sensing material

**Fig. 2:**
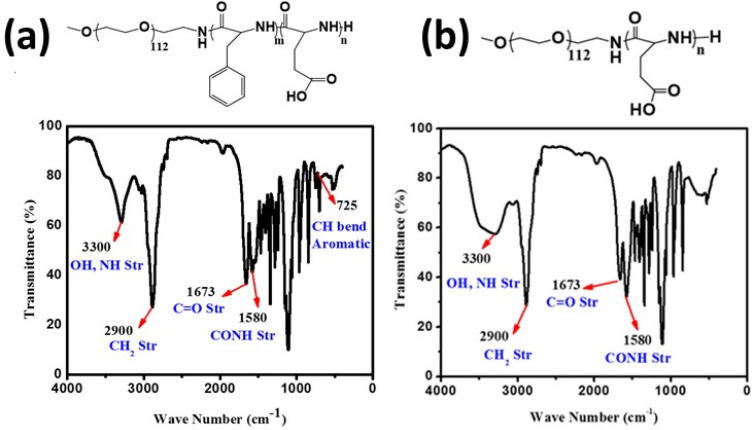
FTIR spectra of (a) mPEG-*b*-PBLG and (b) mPEG-*b*-PLG.

**Fig. 3:**
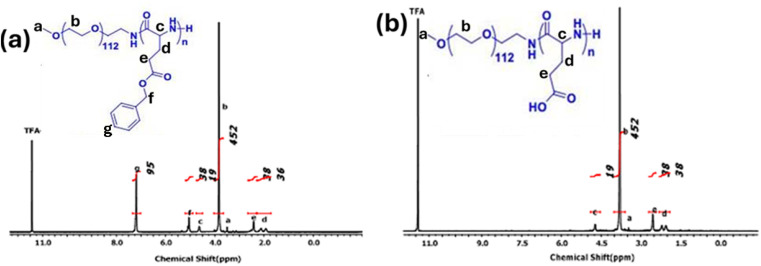
^1^H NMR spectra of (a) mPEG-*b*-PBLG and (b) mPEG-*b*-PLG.

Cadmium oxide quantum dots and polymer methoxy poly(ethylene glycol)-*block*-poly(l-glutamic acid) were used as sensing materials for the detection of glycine. The characterization of the polymer was conducted using FT-IR and proton NMR techniques, as shown in Fig. 2 and 3. The FT-IR results revealed that the polymer was successfully deprotected, as confirmed by the removal of the CH bend aromatic (725 cm^−1^) in the case of mPEG-*b*-PLG. In the same way, a ^1^H NMR spectrum was obtained for mPEG-*b*-PLG, with the exception of the disappearance of peaks at *δ* 7.1 and 5.0 ppm, confirming complete deprotection.^39^

**Fig. 4:**
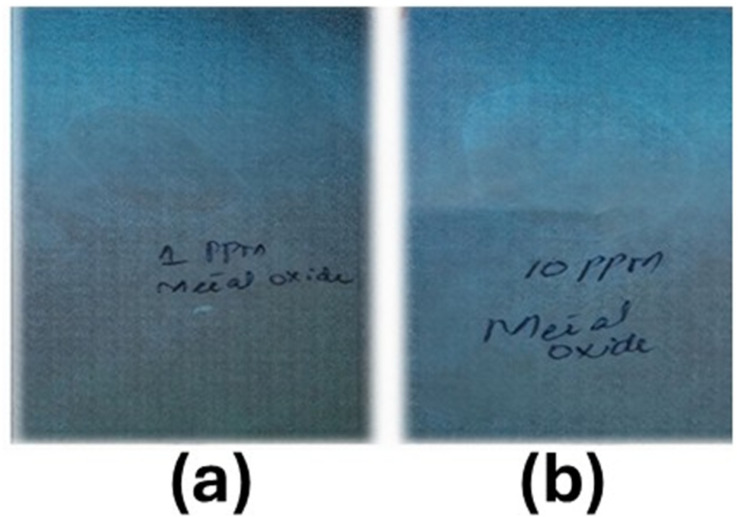
Quantum dots CdOQDs under UV light: (a) 1.0 ppm cadmium oxides; (b) 10.0 ppm cadmium oxides.

The successful synthesis of the quantum dots was confirmed by observing the quantum dots under the UV light of a UV lamp (UVGL-58 handheld UV lamp). A solution of 1.0 and 10.0 ppm quantum dots was observed under a UV lamp, confirming the presence of highly luminescent quantum dots, as shown in Fig. 4. The results demonstrate that the desired quantum dots were successfully synthesized.

### Fabrication and electrochemical behavior of CdONPs/polymer-ePAS

**Fig. 5:**
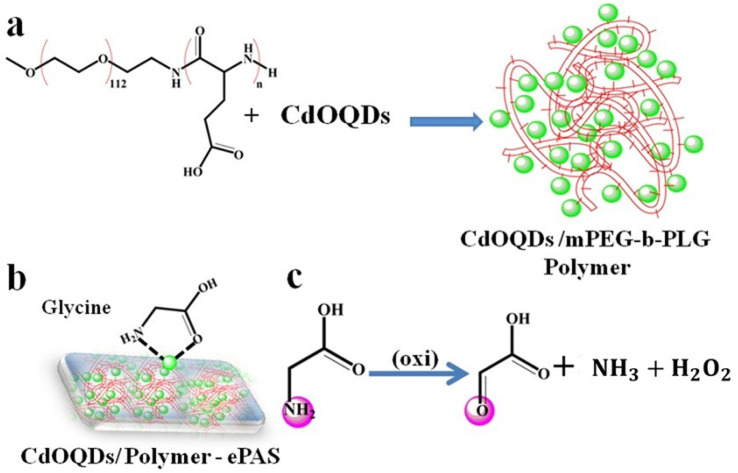
Fabrication and modification of ePAS; polymer-quantum dots (a) modification of e-paper. (b) Interactions of glycine with e-paper. (c) Oxidation of glycine.

Electrochemical sensors for the quantification and detection of glycine are usually developed based on glycine oxidation. The electrochemically based e-paper is illustrated in Fig. 5. As shown in Fig. 5a, the e-paper was modified with polymer/quantum dots, and the interaction between glycine and the e-paper-based sensor is shown in Fig. 5b. The electrochemical oxidation of glycine, which is an aliphatic amino acid, is shown in Fig. 5c. Glycine undergoes oxidation, leading to the formation of glyoxylate, formaldehyde and formic acid, which are possible oxidation products of glycine.

The oxidation of glycine on the surface of an electrochemical sensor requires a higher oxidation potential, which is not possible on a bare carbon electrode because of slow electron transfer reactions. This slow electron transfer process is responsible for very low or no signals at times, which makes the sensors less sensitive with no reproducibility. To make it easy for the electrochemical quantification of glycine, we must introduce an electrocatalyst or redox mediator that has the capability of reducing the oxidation potential, leading to an increase in the electron transfer rate. The first electrocatalyst used in this field consisted of metal complexes, including Ni chelidamic acid, Ni(ii)–baicalein complex, Fe(iii)–Schiff base, Ni(ii) hydroxide and cobalt hydroxide. Ni chelidamic acid is oxidized to a Ni(iii) oxohydride complex when glycine is not present, resulting in an oxidation peak at *ca.* 0.4 V. In the presence of glycine, glycine adsorbed onto the surface and oxidized Ni(ii) centers to Ni(iii). Finally, the Ni(iii) complex oxidizes glycine, being simultaneously reduced to Ni(ii) chelidamic acid and giving rise to an increased intensity of the anodic peak, which is proportional to the glycine concentration in the sample solution. The same procedure was adopted in our e-paper-based sensor, in which the first piece of bare paper interacted with glycine, resulting in no electrochemical reaction. The working electrode was then modified with polymer/quantum dot materials by providing a negative potential from 0 to −0.8 V in a metal ion salt solution. More increase in current was found in the case of the modified electrode surface, which might be due to the catalytic activity of the cadmium oxide quantum dots incorporated within the polymer network leading to the oxidation of glycine. The minimization of the interference effect was observed as confirmed by the absence of oxygen evolution during this process.

### Optimization of the analytical parameters for glycine detection at CdONPs/polymer-ePAS

**Fig. 6:**
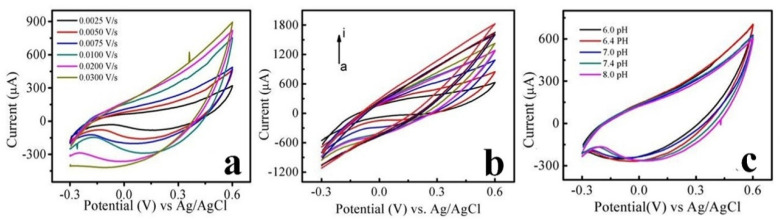
Cyclic voltammetry peaks of glycine at (a) different scan rates (b) different concentrations and (c) at different pH values (experimental conditions: 5 mM glycine, 0.1 mM PBS pH = 8, potential window −0.3 to 0.6 V).

The sensor device efficiency varies with many factors, such as the scan rate, sample concentration, and pH of the liquid substrate. To obtain optimized conditions for the detection of glycine using these sensor devices, cyclic volumetric measurements were performed by varying various factors. The electrochemical oxidation/reduction was monitored by observing the relationship between the peak current and the scan rate of the cyclic voltammogram. The first factor scan rate was investigated for glycine detection (Fig. 6a). To gain insight into the effect of scan rate cyclic voltammetry, glycine was analyzed at different scan rates. The concentration of glycine was maintained at 5.0 mM (the solution was prepared in phosphate buffers of pH 7.0) under the potential window of −0.3 to +0.6 V and at different scan rates starting from 0.03 to 0.0025 V s^−1/2^. Fig. 6a reveals that the peak area and current increase with scan rate. As shown in Fig. 6a, the peak area is directly proportional to the scan rate. The straight-line graph of all peaks obtained at different scan rates was obtained (Fig. S2a and b†), and a straight-line graph (*y* = *mx* + *c*) can be obtained.

Since the protonation of amino groups restricts the formation of bonds between nitrogen and metal ions, it is easy to avoid measurement at physiological pH. The effect of pH on the detection of glycine was investigated to confirm the contribution of protons to electrochemical redox reactions. Different pH values ranging from 6.0 to 8.0 were selected while keeping the scan rate and potential windows optimized. The results of the pH effect are shown in Fig. 6c and S2c.† These graphs show that increasing the pH increased the anodic peak current. It is assumed that at neutral or physiological pH or acidic pH, the amine of the amino acid is protonated. The chance of formation of a bond between nitrogen and metal ions decreases with more protonation, which makes complex formation on the surface of the paper-based sensor difficult.

### Concentration optimization of glycine in CdOQDs/polymer-ePAS

**Fig. 7:**
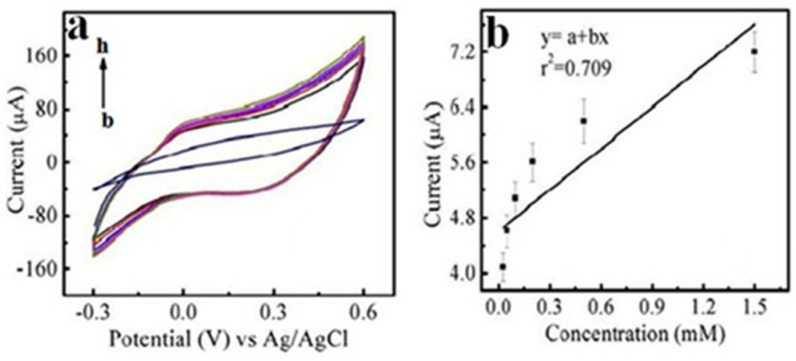
Voltammetry peaks at different concentrations (a) concentration {0.05–1.5 mM} (b) straight line graph between concentration and current, which shows the dependence of peak current on the concentration of samples. Conditions: PBS pH 7.0; scan rate 0.01 V s^−2^; potential window −0.4 to +0.6 V.

Quantum dots of metal oxides can conduct the electrochemical oxidation of various amino acids, including glycine, between 1 and 400 μM at basic pH.^46,47^ In the current investigations, the effect of the concentration of the glycine (blank, 0.05, 0.1, 0.25, 0.5 and 1.5 mM) was obtained using cyclic voltammetry. In this study, the pH of the glycine solution was kept basic (8.0). The scan rate was 0.01 V s^−1/2^ in a potential window from −0.4 to +0.6 V. The results are shown in Fig. 7. The reduction peak exhibits a linear relationship with glycine concentration, as shown in Fig. 7b. The limit of detection was 0.0758 mM or 75.8 μM in the linear range concentration of 0.05–1.5 mM. The limit of quantification was 0.2297 mM or 229.7 μM.

### Stability and reproducibility of CdOQDs/polymer-ePAS

**Fig. 8:**
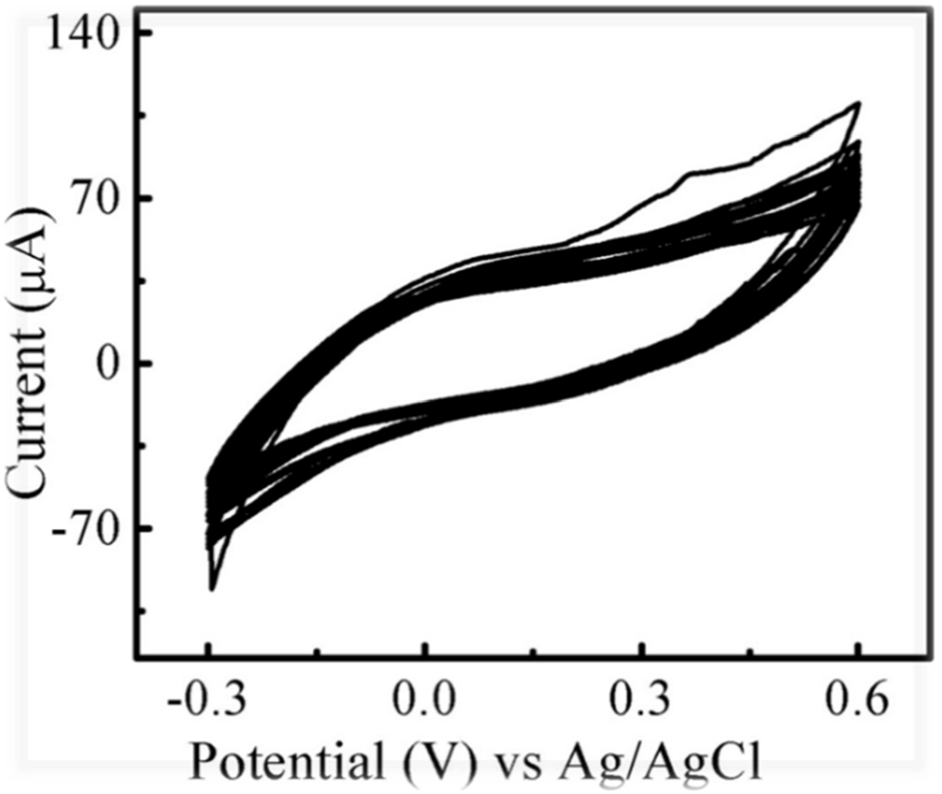
Stability of CdOQDs/polymer-ePAS after 25 cycles. Experimental conditions: (concentration of glycine 5 mM, scan rate 0.01 V s^−2^, 0.1 mM PBS, potential window −0.3 to +0.6 V).

Another feature of point-of-care devices that must be investigated is their stability. In our results (Fig. 8), the developed device was highly stable with more than 25 cycles and a lifetime of more than 2 months. The stability of these devices makes them more economical, eco-friendly, and reproducible. Another important feature of this type of device is its room-temperature storage. This device does not have to be stored in a refrigerator, and it can be easily transported from one place to another.

## Conclusion

In this study, we developed for the first time an innovative electrochemical paper-based analytical sensor (ePAS) for the detection of glycine, a well-known amino acid. The device was fabricated using simple Whatman filter paper as the substrate through a simple wax fabrication method. For precise and reliable electrochemical results, the sensor electrodes were manufactured using carbon and silver/silver chloride inks. To make the device more stable with high electrocatalytic activity, sensing materials were developed from a methoxy poly(ethylene glycol)-*block*-poly(l-glutamic acid) polymer and cadmium oxide quantum dots. These innovative materials accelerate the electron transfer rate with reduced oxidation potential, leading to more accurate and sensitive glycine detection. The sensor achieved a remarkable limit of quantification of 229.7 μM within a linear range of 0.05–1.5 mM, showing high reproducibility over more than 25 operational cycles. The performance of the device was optimized by studying key parameters such as pH, scan rate, and analyte concentration to ensure maximum sensitivity and reliability. Unlike conventional bulky electrochemical setups, the ePAS is lightweight, portable, and cost-effective, making it ideal for point-of-care diagnostics. The environmentally friendly design, which includes biodegradable materials and reusable components, further underscores the potential for large-scale applications. This study highlights the transformative role of nanotechnology and polymer-based materials in electrochemical sensing devices. The developed sensor is not only suitable for glycine detection but also demonstrates versatility for potential applications in detecting other biomolecules and environmental pollutants. Future directions may include improving the long-term stability of the sensor, exploring its multiplexing capabilities, and integrating it with digital platforms for enhanced data acquisition and analysis. The proposed ePAS paves the way for next-generation diagnostic tools that are affordable, efficient, and accessible, catering to the growing demands of personalized and on-site healthcare solutions.

## Ethical statement

This research did not involve any studies with human participants or animals.

## Data availability

The data supporting this article have been included as part of the ESI.†

## Author contributions

Zaheer Ahmad: the main author of the manuscript and contributed to the design and execution of the project. Abdullah K. Alanazi: Helped in thorough analysis and revision of the manuscript as well as contributed in providing financial support for the project from Taif University.

## Conflicts of interest

The authors declare that they have no known competing financial interests or personal relationships that could have appeared to influence the work reported in this paper.

## Supplementary Material

RA-015-D5RA01870F-s001
