# Supplementary material for: Polymer thin film-embedded metal oxide-modified electrochemical paper-based sensor for glycine detection
Source: RSC Adv. 2025 May 12;15(20):15630–8. doi: 10.1039/d5ra01870f (PMC12067406; doi:10.1039/d5ra01870f)
Supplement: RA-015-D5RA01870F-s001 [file RA-015-D5RA01870F-s001.pdf]

Supplementary Information (SI) for RSC Advances.
This journal is © The Royal Society of Chemistry 2025

Supplementary Informations

Polymer Thin Film Embedded Metal Oxide Modified Electrochemical Paper Based Sensor for Glycine detection

[1,2] Zaheer Ahmad and [3]Abdullah K. Alanazi

[1]Department of Chemistry, GPGC Timergara Dir Lower Khyber Pakhtunkhwa, Pakistan

[2]Higher Education, Archives and Libraries Department Government of Khyber Pakhtunkhwa, Pakistan

[3]Department of Chemistry, College of Science, Taif University, P.O. Box 11099, Taif 21944, Saudi Arabia

[*1,2]saubanzaheer@gmail.com https://orcid.org/0000-0002-9837-8647

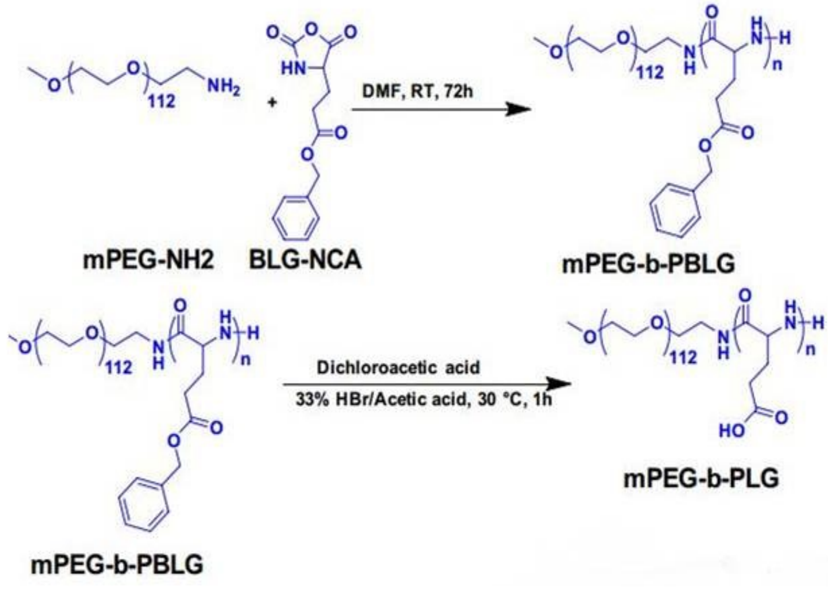

Scheme S1: Scheme for the synthesis of mPEG-block-PLG

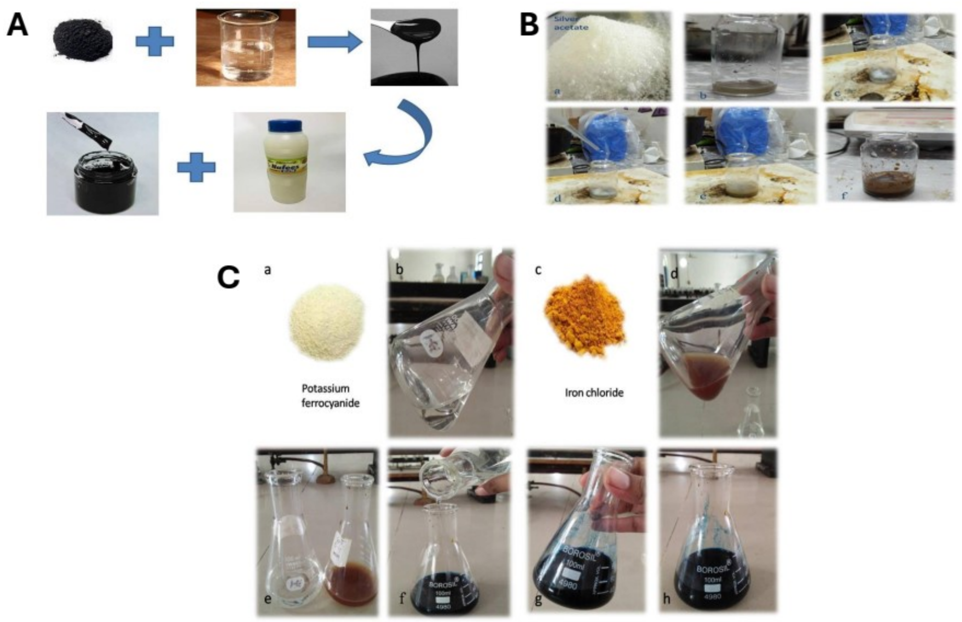

Figure .S1. Preparation of inks for electrode fabrication at paper**: A.** Carbon Ink, **B.** Silver ink as Ag/AgCl Electrode, **C.** Prussian Blue as mediator

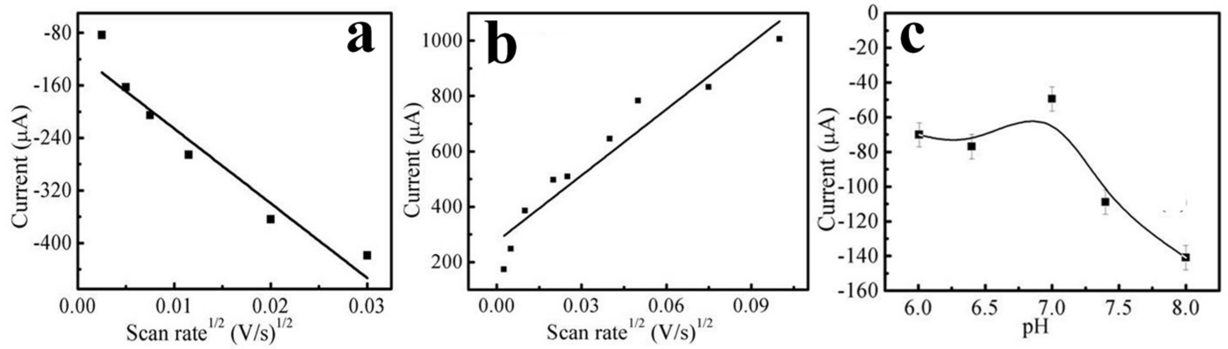

Figure. S2. Liner Electrochemical behavior for concentration optimization of glycine at CdONPs/ polymer paper-based sensor at different scan rates (**a and b**) and pH (**c**) (concentration of glycine 5 mmol, buffer 0.1 mol pH=7.0, and potential window from -0.3 to + 0.6).
